# Artificial Intelligence: Does Consciousness Matter?

**DOI:** 10.3389/fpsyg.2019.01535

**Published:** 2019-07-02

**Authors:** Elisabeth Hildt

**Affiliations:** Center for the Study of Ethics in the Professions, Illinois Institute of Technology, Chicago, IL, United States

**Keywords:** consciousness, artificial intelligence, ethics, robots, human-robot interaction

Consciousness plays an important role in debates around the mind-body problem, the controversy over strong vs. weak artificial intelligence (AI), and bioethics. Strikingly, however, it is not prominent in current debates on ethical aspects of AI and robotics. This text explores this lack and makes two claims: We need to talk more about artificial consciousness and we need to talk more about the lack of consciousness in current robots and AI.

## Can Machines Have Consciousness?

The question of whether machines can have consciousness is not new, with proponents of strong artificial intelligence (strong AI) and weak AI having exchanged philosophical arguments for a considerable period of time. John R. Searle, albeit being critical toward strong AI, characterized strong AI as assuming that “…the appropriately programmed computer really is a mind, in the sense that computers given the right programs can be literally said to understand and have cognitive states” (Searle, 1980, p. 417). In contrast, weak AI assumes that machines do not have consciousness, mind and sentience but only simulate thought and understanding.

When thinking about artificial consciousness, we face several problems (Manzotti and Chella, 2018). Most fundamentally, there is the difficulty to explain consciousness, to explain how subjectivity can emerge from matter—often called the “hard problem of consciousness” (Chalmers, 1996). In addition, our understanding of human consciousness is shaped by our own phenomenal experience. Whereas, we know about human consciousness from the first-person perspective, artificial consciousness will only be accessible to us from the third-person perspective. Related to this is the question of how to know whether a machine has consciousness.

A basic assumption for artificial consciousness is that it be found in the physical world of machines and robots (Manzotti and Chella, 2018). Furthermore, any definition of artificial consciousness given by humans will have to be made from the third-person perspective, without relying on phenomenal consciousness.

One strategy is to avoid a narrow definition of machine consciousness, or to avoid giving a definition at all. An example of this strategy is given by David Levy (Levy, 2009, p. 210) who prefers to take a pragmatic view according to which it is sufficient to have a general agreement about what we mean by consciousness and suggests “let us simply use the word and get on with it.”

Other authors focus on self-awareness. With regard to self-aware robots, Chatila et al. (2018, p. 1) consider relevant: “… the underlying principles and methods that would enable robots to understand their environment, to be cognizant of what they do, to take appropriate and timely initiatives, to learn from their own experience and to show that they know that they have learned and how.” In contrast, Kinouchi and Mackin focus on adaptation at the system-level (Kinouchi and Mackin, 2018, p. 1), “Consciousness is regarded as a function for effective adaptation at the system-level, based on matching and organizing the individual results of the underlying parallel-processing units. This consciousness is assumed to correspond to how our mind is “aware” when making our moment to moment decisions in our daily life.”

In order to solve questions specific to artificial consciousness, it is helpful to consider the philosophical reflection around consciousness, which focuses on human (and animal) consciousness. There are many concepts of consciousness. Normally, we distinguish between (a) a conscious entity, i.e., an entity that is sentient, wakeful, has self-consciousness and subjective qualitative experiences, (b) being conscious of something, for example a rose, and (c) conscious mental states, i.e., mental states an entity is aware of being in, such as being aware of smelling a rose (Van Gulick, 2018; Gennaro, 2019).

For the discussion of artificial consciousness, Ned Block's distinction between phenomenal consciousness and access consciousness proves to be particularly helpful (Block, 1995). Whereas, phenomenal consciousness relates to the experience, to what it is like to be in a conscious mental state, access consciousness refers to a mental state's availability for use by the organism, for example in reasoning and guiding behavior, and describes how a mental state is related with other mental states. The debate on artificial consciousness would clearly benefit from focusing on access consciousness.

Dehaene et al. (2017) distinguish two essential dimensions of conscious computation: global availability (C1) and self-monitoring (C2). Global availability, which they characterize as information being globally available to the organism, resembles Ned Block's access consciousness (Block, 1995). Self-monitoring (C2), which they consider as corresponding to introspection, “refers to a self-referential relationship in which the cognitive system is able to monitor its own processing and obtain information about itself” (pp. 486–487).

As the examples of approaches to define artificial consciousness given above show, different authors stress different aspects. There clearly is room for more reflection and research on what third-person definitions of artificial consciousness could look like.

## Artificial Consciousness and Human-Robot Interaction

Overall, researchers broadly agree that current machines and robots are not conscious—in spite of a huge amount of science fiction depictions that seem to suggest otherwise. In a survey with 184 students, however, the answers to the question “Do you believe that contemporary electronic computers are conscious?” were: No: 82%; Uncertain: 15%; Yes: 3% (Reggia et al., 2015). Remarkably, the question in the survey was about “contemporary electronic computers,” and not about AI or robots.

Consciousness-related questions may be expected to arise most easily with social robots and human-robot social interaction (Sheridan, 2016). According to a definition given by Kate Darling (Darling, 2012, p. 2), a social robot “is a physically embodied, autonomous agent that communicates and interacts with humans on a social level.” Examples of social robots include MIT's Kismet, Aldebaran NAO, and the humanoid social robot Sophia by Hanson Robotics.

Social robots have several characteristics that make them special for humans: They are capable of limited decision-making and learning, can exhibit behavior, and interact with people. In addition, capabilities like nonverbal immediacy of robot social behavior (Kennedy et al., 2017), speech recognition and verbal communication (Grigore et al., 2016), facial expression, and a perceived “personality” of robots (Hendriks et al., 2011), play important roles in how humans respond to robots.

Consequently, humans tend to develop unidirectional emotional bonds with robots, project lifelike qualities, attribute human characteristics (anthropomorphizing), and ascribe intentions to social robots (Scheutz, 2011; Darling, 2012; Gunkel, 2018). A typical example, if not a culmination of this tendency, can be seen in the social humanoid robot Sophia being granted Saudi-Arabian citizenship in 2017 (Katz, 2017).

All of this raises questions concerning the status of robots, and how to respond to and interact with social robots (Gunkel, 2018). Are social robots mere things? Or are social robots quasi-agents or quasi-persons (Peter Asaro)? Socially interactive others? Quasi-others? Should robots have rights?

Even though there is a general agreement that current robots do not have sentience or consciousness, some authors (such as Coeckelbergh, 2010; Darling, 2012; Gunkel, 2018) have argued in favor of ascribing rights to robots. For example, based on research on violent behavior toward robots, Kate Darling argues that it is in line with our social values to treat robots more like pets than like mere things.

While the exact arguments in favor of ascribing rights to robots differ, what is common to these positions is that they focus on the social roles humans ascribe to robots, the relationships and emotional bonds humans build with robots, or on the social context in which humans interact with robots. They do not ascribe status based on robot capabilities but argue in favor of rights based on the role robots play for human beings.

There is a fundamental problem with this “social roles” approach, however. The suggestions it makes on how to interact with robots are not consistent with the way we interact with human beings (see also Katz, 2017). The “social roles” approach, transferred to human beings, would claim that a human being's value or rights depend strongly on his or her social roles or the interests of others. This claim would be in contradiction to the generally held view that human beings have moral status independent of their social roles. From this perspective, an entity has moral status “…if and only if it or its interests morally matter to some degree for the entity's own sake” (Jaworska and Tannenbaum, 2018).

For the ascription of status and rights to human beings, personhood is central. The concept of a person involves a number of capabilities and central themes such as rationality; consciousness; personal stance (the attitude taken toward an entity); capability of reciprocating the personal stance; verbal communication; and self-consciousness (Dennett, 1976). Daniel C. Dennett considers all of these as necessary conditions of moral personhood.

In contrast, according to the “social roles” approach, rights are being ascribed not on the basis of a robot's moral status or capabilities, but on the basis of the social roles it plays for others. This explains why consciousness does not matter for this position. For it is not plausible to claim that current robots matter morally for their own sake as long as they lack characteristics such as sentience or consciousness.

This may change in the future, however. Then it may be plausible to think about a concept of “robothood” and ascribe moral status to these future robots, based on their capabilities. There is already an interesting and controversial discussion going on about ascribing legal personhood to robots (Bryson et al., 2017; Solaiman, 2017). For the debate on the moral and legal status of robots, but also for the broader question of how to respond to and interact with machines, a better understanding of artificial consciousness, artificial rationality, artificial sentience, and similar concepts is needed. We need to talk more about artificial consciousness and the lack of consciousness in current AI and robots. In this, focusing on third-person definitions of artificial consciousness and access consciousness will prove particularly helpful.

## Author Contributions

The author confirms being the sole contributor of this work and has approved it for publication.

### Conflict of Interest Statement

The author declares that the research was conducted in the absence of any commercial or financial relationships that could be construed as a potential conflict of interest.
